# CRISPR/Cas9-induced β-carotene hydroxylase mutation in *Dunaliella salina* CCAP19/18

**DOI:** 10.1186/s13568-021-01242-4

**Published:** 2021-06-07

**Authors:** Lina Hu, Shuying Feng, Gaofeng Liang, Jingxia Du, Aifang Li, Chunling Niu

**Affiliations:** 1 School of Basic Medical Sciences, Henan University of Science and Technology, No. 263 Kaiyuan Avenue, Luoyang, 471023 Henan China; 2 Medical College, Henan University of Chinese Medicine, Zhengzhou, 450046 Henan China; 3 Academy of Chinese Medicine Sciences, Henan University of Chinese Medicine, Zhengzhou, 450046 Henan China

**Keywords:** *D. salina*, CRISPR/Cas9, β-carotene hydroxylase, Knockout, Mutant

## Abstract

*Dunaliella salina* (*D. salina*) has been exploited as a novel expression system for the field of genetic engineering. However, owing to the low or inconsistent expression of target proteins, it has been greatly restricted to practical production of recombinant proteins. Since the accurate gene editing function of clustered regularly interspaced short palindromic repeat (CRISPR)/Cas system, β-carotene hydroxylase gene was chosen as an example to explore *D. salina* application with the purpose of improving expression level of foreign genes. In this paper, based on pKSE401 backbone, three CRISPR/Cas9 binary vectors were constructed to targeting exon 1 and 3 of the β-carotene hydroxylase of *D. salina* CCAP19/18 (Dschyb). *D. salina* mutants were obtained by salt gradient transformation method, and the expression of Dschyb gene were identified through real-time fluorescent quantitative PCR. Moreover, carotenoids content was analyzed by high-performance liquid chromatography at different time points after high intensity treatment. Compared with wild type strains, the β-carotene levels of mutants showed a significant increase, nearly up to 1.4 μg/ml, and the levels of zeaxanthin decreased to various degrees in mutants. All the results provide a compelling evidence for targeted gene editing in *D. salina*. This study gave a first successful gene editing of *D. salina* which has a very important practical significance for increasing carotene yield and meeting realistic industry demand. Furthermore, it provides an approach to overcome the current obstacles of *D. salina*, and then gives a strong tool to facilitates the development and application of *D. salina* system.

## Introduction

Presently, *Dunaliella salina* (*D. salina*) is the best commercial source of natural β-carotene. According to the optimized condition, β-carotene could accumulate up to 32.0 mg/L (Xi et al. 2020). And now, *D. salina* has been used as a huge commercial sources of carotenoids, such as phytoene, phytofluene, lutein, and zeaxanthin (Xu et al. 2018; Saha et al. 2018; Liang et al. 2019). Not only that, the raising market demand for natural pigments increased the efforts to enhance production of carotenoids from biological sources. Although several studies have addressed the effect of different culture conditions on β-carotene content in *D. salina*, its accumulation leads to reduced growth rates, including extreme temperatures, high salinity, and nitrogen limitation (Xu and Harvey 2019). So, molecular approaches have shown great potentials to enhance the accumulation of bio-products, such as metabolic engineering, transcriptional engineering, and gene disruption strategies (Wichuk et al. 2014; Zhang et al. 2018).

The clustered regularly interspaced short palindromic repeat (CRISPR) technology is a versatile tool to perform genome editing in different organisms ranging from prokaryote to eucaryote. And now, CRISPR/Cas system has been used as an enormously powerful tool in the drug discovery, disease treatment, pollution control, and other fields (Fellmann et al. 2017; Khadempar et al. 2019; Feng et al. 2020). Thus, this system offers an excellent integration point for development of *D. salina* bioreactor. Competent CRISPR-based genome editing techniques have been reported in several microalgal species, such as enhancing of lipid content, and improving of biomass production (Liang et al. 2019). Compare with the traditional transgenic methods, CRISPR/Cas system has advantages of easy design, high-efficiency, and capability of multiplex genome editing (Sternberg et al. 2014).

β-Carotene hydroxylase is a key enzyme in the pathway of carotenoid biosynthesis in plants, which catalyse the conversion of β-carotene to zeaxanthin for carotenoid biosynthetic pathway (Lamers et al. 2008). In theory, blocking or silencing this enzyme can increase the content of β-carotene in *D. salina* cells. For instance, RNA interference technology has been used to silence β-carotene hydroxylase of maize endosperm and potatoes, which successfully increased the carotene content (Berman et al. 2017; Kim et al. 2012). To test our hypothesis, we attempted to knockout β-carotene hydroxylase of *D. salina* (Dschyb) gene with CRISPR/Cas9 system. The results demonstrated that the specific-mutants of Dschyb have been successfully created by CRISPR technology*.* This is the first report of gene editing in *D. salina* with the CRISPR/Cas system. It not only can facilitate the development and application of *D. salina* system, but also give a strong tool to overcome the current obstacles of *D. salina*.

## Materials and methods

### Algal strain and culture conditions

*D. salina *CCAP19/18 D Kessler were purchased from the Guangyu Biotech Co., Ltd (Wenzhou, China), and cultured in the modified PKS medium, which comprising 1.5 M NaCl, 10 mM KNO_3_, 50 mM NaHCO_3_, 5 mM MgSO_4_.7H_2_O, 0.4 mM KH_2_PO_4_, 2 μM FeCl_3_∙6H_2_O, 5 μM EDTA, 7 μM MnCl_2_∙4H_2_O, 1 μM CuCl_2_∙2H_2_O, 1 μM ZnCl_2_, 1 μM CoCl_2_∙6H_2_O, 1 μM (NH_4_) Mo_7_O_24_.4H_2_O, 185 μM H_3_Bo_3_, 0.2 mM CaCl_2_, at 26℃ with a 12 h-light/day under light intensity of 4000 Lux. The pKSE401 vector was a gift from Professor Gongyao Shi of Zhengzhou University. In previous studies, pKSE401 vector has been successfully applied into *Arabidopsis* (Xing et al. 2014) and *Brassica napus* (*B. napus*) (Yang et al. 2017) to generate mutants with a high efficiency. zCAS9-NLS was under control of two constitutive 35S promoters, and the sgRNA scaffolds expression was driven by *Arabidopsis* U6-26 promoter.

### Vector construction and *D. salina* transformation

**Fig. 1:**
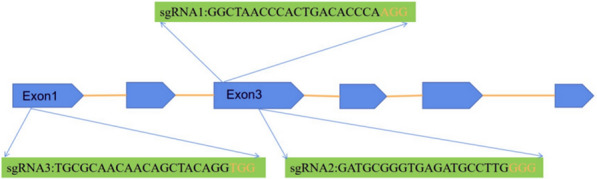
Schematic diagram of three sgRNA targeting sites for Dschyb locus. Blue module represents exon; Orange line represents intron; Orange marked sequence is PAM site

We manually search for 23 bp target sites (5'-N 20 NGG-3') within exons of genomic DNA sequences of Dschyb (GenBank: KX096216.1) on the website of http://crispr.dbcls.jp/. Based on the optimization principles (Nymark et al. 2016), three single guide RNA (sgRNA) sequences were designed to introduce two *Bsa* I flanking sites and named in sgRNA1-F, sgRNA2-F and sgRNA3-F, respectively (Table 1). Golden gate cloning was used to assemble sgRNA, which allows to obtain nearly one hundred percent correct recombinant plasmids (Vecchione et al. 2019). For assembly of sgRNA, equal volumes of 100 μM oligos 1 and 2 were mixed and then incubated at 80℃ for 10 min. After that, the mixture was cooled slowly to room temperature, resulting in a double-stranded insert with 4-nt 5′ overhangs at both ends. Annealed double-stranded oligonucleotides together with pKSE401 was used to set up restriction-ligation reactions using *Bsa* I and T4 ligase (NEB, Ipswich, USA). The reaction was incubated orderly in a thermocycler for 2 h at 37℃, 5 min at 50℃ and 10 min at 80℃. Consequently, the constructed plasmids were named as pKSE401-sgRNA1, pKSE401-sgRNA2 and pKSE401-sgRNA3, which correspond to the regions in exon 3 and 1 of Dschyb, respectively (Fig. 1). Using these constructed plasmids, *D. salina* cells were transformed separately with salt gradients method (Feng et al. 2020). After 2–3 days transformation, cells were cultured in fresh medium with 700 µg/mL G418 in a 12-well plate at 26℃ light incubator. And then, transformed cells were plated on the solid antibiotic-free medium (0.8% agarose, w/v) to obtain single colonies. During 10 days culture, culture dishes were inverted and placed in an illuminated incubator with 12 h light / 12 h dark (26 °C) photo-period. Finally, the positive colonies were detected by polymerase chain reaction (PCR) amplification and gene sequencing.

**Table 1:** The primers used for different vectors construction

Primer name	Base sequence (5’-3’)	Vector name
sgRNA1-F	ATTGGGCTAACCCACTGACACCCA	pKSE401-sgRNA1
sgRNA1-R	AAACTGGGTGTCAGTGGGTTAGCC	
sgRNA2-F	ATTGGATGCGGGTGAGATGCCTTG	pKSE401-sgRNA2
sgRNA2-R	AAACCAAGGCATCTCACCCGCATC	
sgRNA3-F	ATTGTGCGCAACAACAGCTACAGG	pKSE401-sgRNA3
sgRNA3-R	AAACCCTGTAGCTGTTGTTGCGCA

### PCR identification of mutants

The single algae colonies were picked with a 10 µL inoculation loops and re-suspended in fresh medium. Another portion of the same single colony was directly used for single algae colony PCR with the Phire Plant Direct PCR Master Mix kit (Thermo Fisher Scientific, Shanghai, China). Briefly, a 0.5 mm single algae colony was picked from the agar plate using a pipette. The samples were placed directly into 50 µL PCR reactions. Reactions were run following the cycling conditions: 5 min at 98 ℃, then 35 cycles of 5 s at 98 ℃, 5 s at 58.4 ℃, 40 s at 72 ℃, and a final extension of 1 min at 72 ℃. The PCR products were analyzed by 1% agarose gel electrophoresis. Next, Mutants were screened by analyzing the size of PCR products. Sanger sequencing reactions of PCR products were carried out by Sangon Biotech Co., Ltd (Shanghai, China). Finally, DNA sequences were analyzed with the snapgene software.

### sgRNA transcription in vitro and cleavage assay

Using pKSE401 as the template, forwards primers were designed for adding the T7 promoter sequence (TTAATACGACTCACTATAGG) into sgRNA loop by PCR. T7 sgRNA-R-common was used as the reverse primer to obtain the final DNA fragment for transcription in vitro. PCR was carried out using 2X high fidelity PCR master mix (Sangon, Shanghai, China) as follows: initial denaturation at 95 ℃ for 30 s, 35 cycles of 95 ℃ for 15 s, 58 ℃ for 15 s and 72 ℃ for 20 s, a final extension at 72 ℃ for 5 min, and termination at 4 ℃. The T7-sgRNA primer sequence are summarized in Table 2, which named T7-sgRNA-F1, T7-sgRNA-F2, and T7-sgRNA-F3, respectively. The corresponding DNA were transcribed in vitro with the HiScribe T7 high yield RNA synthesis kit (NEB, Ipswich, USA). Next, sgRNAs (T7-sgRNA1, T7-sgRNA2 and T7-sgRNA3) for targeting Dschyb were produced with the transcriptions in vitro.

**Table 2:** The primers used for sgRNA transcription in vitro and target genes

Primer name	Base sequence (5’-3’)	Purpose
T7-sgRNA-F1	TTAATACGACTCACTATAGGCTAACCCACT GACACCCAGTTTTAGAGCTAGAAATAGC	Transcription in vitro
T7-sgRNA-F2	TTAATACGACTCACTATAGGATGCGGGTGA GATGCCTTGGTTTTAGAGCTAGAAATAGC	Transcription in vitro
T7-sgRNA-F3	TTAATACGACTCACTATAGGTGCGCAACAA CAGCTACAGGGTTTTAGAGCTAGAAATAGC	Transcription in vitro
T7 sgRNA-R-common	AAAAGCACCGACTCGGTGCC	Transcription in vitro, colony PCR
CHY-F	AAGGTCAACACAAGGGAACGA	Target gene Dschyb
CHY-R	GTTTGGTGTCAGGAAGCCGT	

Before cleavage assay, total RNAs were purified using Trizol (Cwbio, Taizhou, China) and finally dissolved in RNase free water. Purified RNA was quantified by spectrometry for further use. To obtain the RNP complex harbouring pre-assembled Cas effector protein and the targeting sgRNA, equimolar amount of Cas protein (YSY, NanJing, China) and sgRNA were incubated in a sterile 1.5 mL eppendorf tube at 37 ℃ for 15 min along with 3 µL 10X Cas buffer and milliQ water to a final volume of 30 µL. After 15 min incubation, 400 ng of target DNA was added into the reaction mixture and incubated again for 1 h. And then, Cas9 nuclease was inactivated at 65 °C for 10 min. The target DNA was amplified from the host genomic DNA with the high fidelity PCR master mix (Sangon, Shanghai, China) using primers CHY-F and CHY-R (Table 2). After that, PCR fragments were analyzed by 1% agarose gel electrophoresis. Simultaneously, this assay was performed with individual ribonucleoprotein (RNP) complex or with different duplex combinations.

### Comparison of β-carotene hydroxylase level in *D. salina* and its pigment analysis

Usually, carotenoids accumulated with increasing light emitting diode (LED) light intensity. So, Cells were grown under 12/12 light/dark (L/D) with 4000 Lux m^−2^ s^−1^ supplied by LED light to exponential growth phase and then exposed continuously to 6000 Lux m^−2^ s^−1^ LED light. After further growth for 24 h in white LED light, cultures were used to monitor the changes in the Dschyb mRNA levels and cellular carotenoids. After that, expression levels of Dschyb in different strains were examined by real-time fluorescent quantitative PCR (RT-qPCR). RT-qPCR reaction was conducted in 96-well plates containing cDNA template and gene specific primers (Table 3). At the same time, the housekeeping gene β-actin was used as an internal standard. Total RNA and cDNA amounts were determined with the NanoDrop One spectrophotometer (Thermo Scientific, Madison, USA). Subsequently, gene expression level was calculated by 2^−∆∆Ct^ method with ABI 7500 manager program. On the other hand, biomass was harvested and extracted for high-performance liquid chromatography (HPLC) analysis as described by Xu et al. with slight modification (Xu and Harvey 2019). Using a Hypersil GOLD™ C-18 reverse phase (250 × 4.6 mm, 5 μm particle size) HPLC column (Thermo Scientific,Waltham, USA), β-carotene and zeaxanthin amounts were analyzed with an isocratic solvent system (90% methanol:10% acetonitrile) at 37℃ for 20 min and flow rate of 1 mL/min at a pressure of 78 bar. Standards for carotenoids (β-carotene and zeaxanthin) were obtained from Yuanye Biotech Co., Ltd (Shanghai, China) and dissolved in mobile phase to generate standard curves. Their quantities were performed at wavelengths of 450 nm with diode array detector (DAD) detector. Pigments were extracted from the 1 mL culture biomass using 1 mL of 80% (V/V) acetone, and placed at minus 20 degrees overnight. Before HPLC analysis, clarified samples were filtered with 0.22 µm filter into amber vials.

**Table 3:** The primers for RT-qPCR

Primer name	Base sequence (5’-3’)	Purpose
B-CHY-F	GGAGCAGCAATTTTACAAGC	Dschyb RT-qPCR
B-CHY-R	CACTCCTCCAGCCACAAG	
β-actin-F	CGACCGCATGAGCAAAGAGA	β-actin RT-qPCR
β-actin-R	CGCTCTCGTCGTACTCTGAC	

### Statistical analysis

In present study, differences of related gene expression between wild-type (WT) and mutant strain were statistically analyzed using one-way analysis of variance. The statistical significance of differences was determined using t-test and accepted when *P* value was less than 0.05. Data were plotted using GraphPad Prism software version 8.0 (GraphPad Software, San Diego, USA).

## Results

### sgRNAs cleavage efficacy assay

**Fig. 2:**
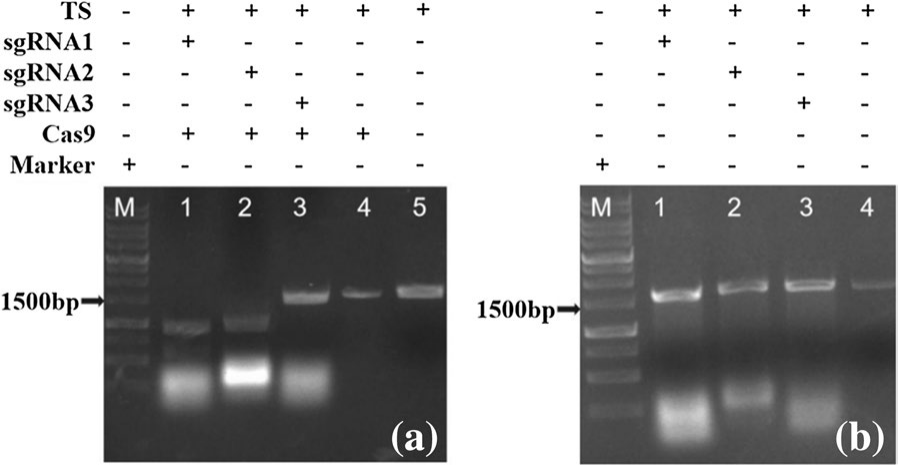
Analysis of sgRNA cleavage activity for target DNA. **a** Cleavage results of three sgRNA-Cas9 complexes to target DNA. **b** Cleavage results of single three sgRNAs to target DNA. TS: PCR products of target DNA; sgRNA: transcribed RNAs in vitro; Cas9: Cas9 protein; + : The plus represents the component in reaction system. −: The minus shows no this component in reaction system

Using primers of Dschyb (named as CHY-F and CHY-R), 1643 bp sgRNA-targeted fragments were amplified by PCR with WT *D. salina* genomes. After cleavage efficacy testing of three Cas9 RNP complexes, the results demonstrated that sgRNA1-Cas9 and sgRNA2-Cas9 complexes showed a 100% cleavage efficiency and yielded a band of expected size. As shown in Fig. 2, the former complex cleaved the target DNA fragment into a 646 bp and a 997 bp lane (Fig. 2 a1), while the latter complex cleaved the target template into a 1057 bp and a 586 bp lane (Fig. 2 a2). But the third complex (sgRNA3-Cas9) failed to cleave the target fragment which offers the third sgRNA is inactive (Fig. 2 a3). When using sgRNA and Cas9 protein alone, it was found that neither sgRNA nor Cas9 protein had cleavage activity to the target fragment. The complex becomes cleavage active only after they bound to each other. The reason mainly results from the different efficiency to CG content, presence of a guanine proximal to the PAM site, and the secondary structure of RNA fold (Jiang et al. 2014).

### *D. salina* transformation and identification of mutants

**Fig. 3:**
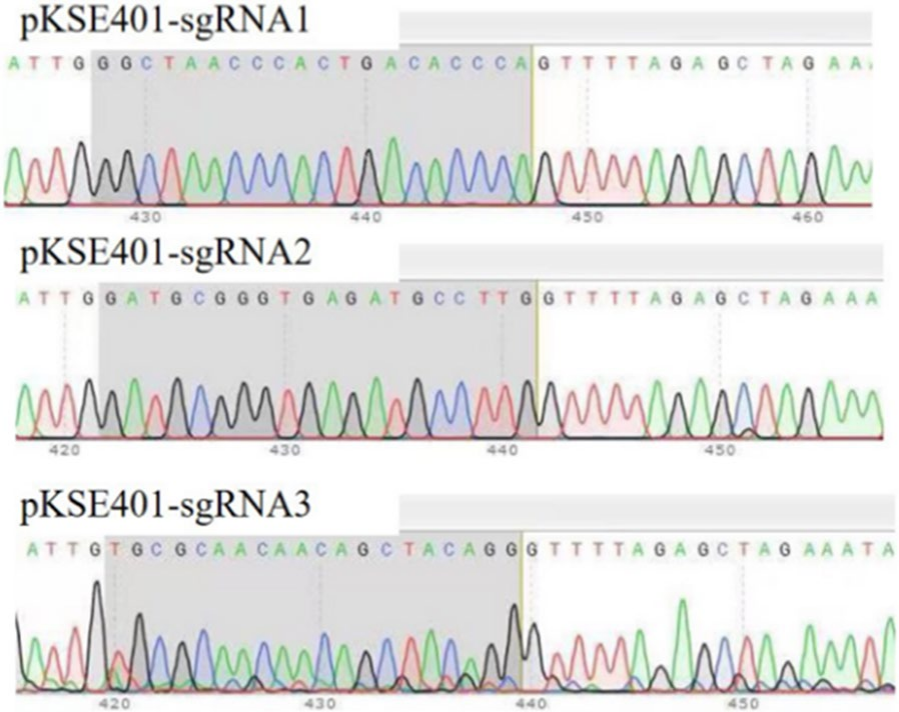
Sequencing identification of three sgRNA sequences (Gray parts represent the sgRNA base sequences)

**Fig. 4:**
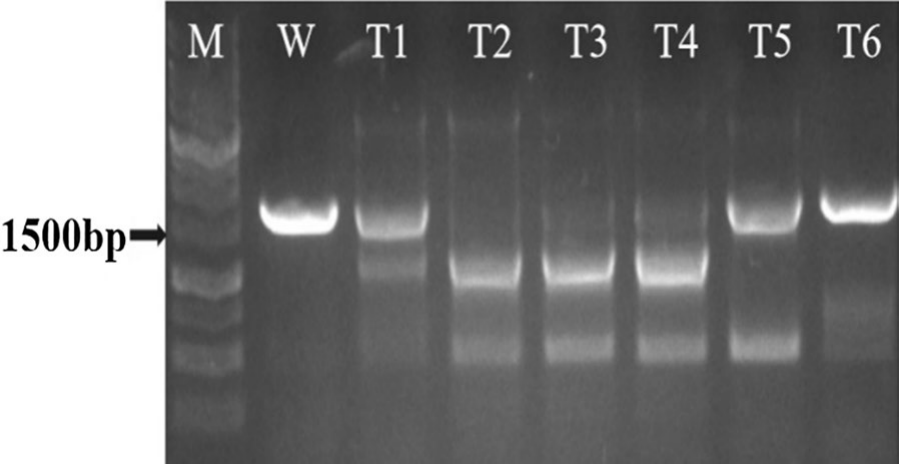
The results for single algae colony PCR. W: Wild type *D. salina* strain; T1-6: Transformed *D. salina* lines

According to sgRNA preferred principle, three vectors were successfully constructed with conserved domains of Dschyb gene and confirmed by sequencing (Fig. 3). Based on test results, the effective pKSE401-sgRNA_1_ and pKSE401-sgRNA_2_ plasmids were used to transform the *D. salina* cells. Meanwhile, to increase the editing rate, the mixture of pKSE401-sgRNA_1_ and pKSE401-sgRNA_2_ has been used to transformed the *D. salina* cells. After transformation, six colonies were randomly selected and the Dschyb genes of each sample were amplified by colony PCR. As shown in Fig. 4, PCR products of three mutant strains showed a fragment of about 1 kb in length (Fig. 4, lane T2-4) which shorter than that of WT *D. salina* (Fig. 4, lane W, 1.6 kb). After gene sequencing, PCR products displayed overlapped peaks in the sequencing chromatography. The WT line and three isolates without target bands were picked for further analysis.

### Expression of Dschyb gene in mutants

**Fig. 5:**
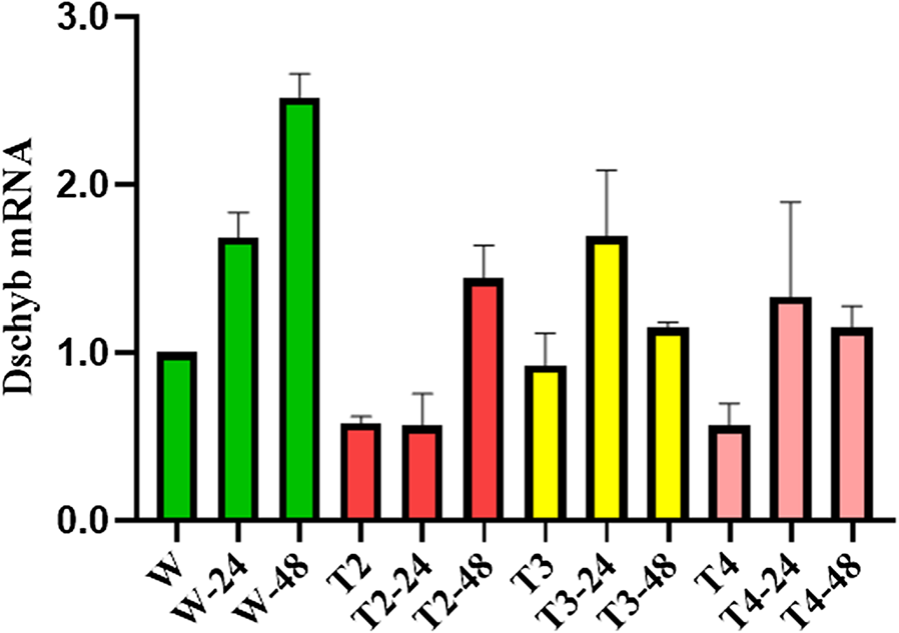
Relative expression level of Dschyb gene at different times after exposure to high intensity LED light (Values represented as mean ± SD (n = 3)). W, W-24, W-48: The mRNA expression levels of Dschyb in WT strain which exposure to high light for 0 h, 24 h, and 48 h, respectively; T, T-24, T-48: The expression level of Dschyb in mutant which exposure to high light for 0 h, 24 h, and 48 h, respectively

Through RT-qPCR analysis, relative expression levels of Dschyb mRNA in mutants were compared with those of WT. The results showed that, compared with the WT *D. salina*, the Dschyb mRNA levels in T2, T3 and T4 lines were reduced to 58%, 92.5%, and 56.5%, respectively (Fig. 5). In view of this, the expression of the edited Dschyb gene was significantly reduced in mutants. Unfortunately, none of the phenotype showed the complete silencing of Dschyb gene in *D. salina*. Under the light exposure, transcriptional level of Dschyb was up-regulated at 24 h and reached the highest level at 48 h (*P* < 0.01) in the WT cells. After constant exposure to strong light conditions, Dschyb mRNA of T3 and T4 lines reached the highest level at 24 h, while T2 was noted the highest level at 48 h. Nevertheless, the levels of Dschyb mRNA of mutant showed significantly decrease than that of WT cells after the high light stress.

### Determination of carotenoid profile in mutants

**Fig. 6:**
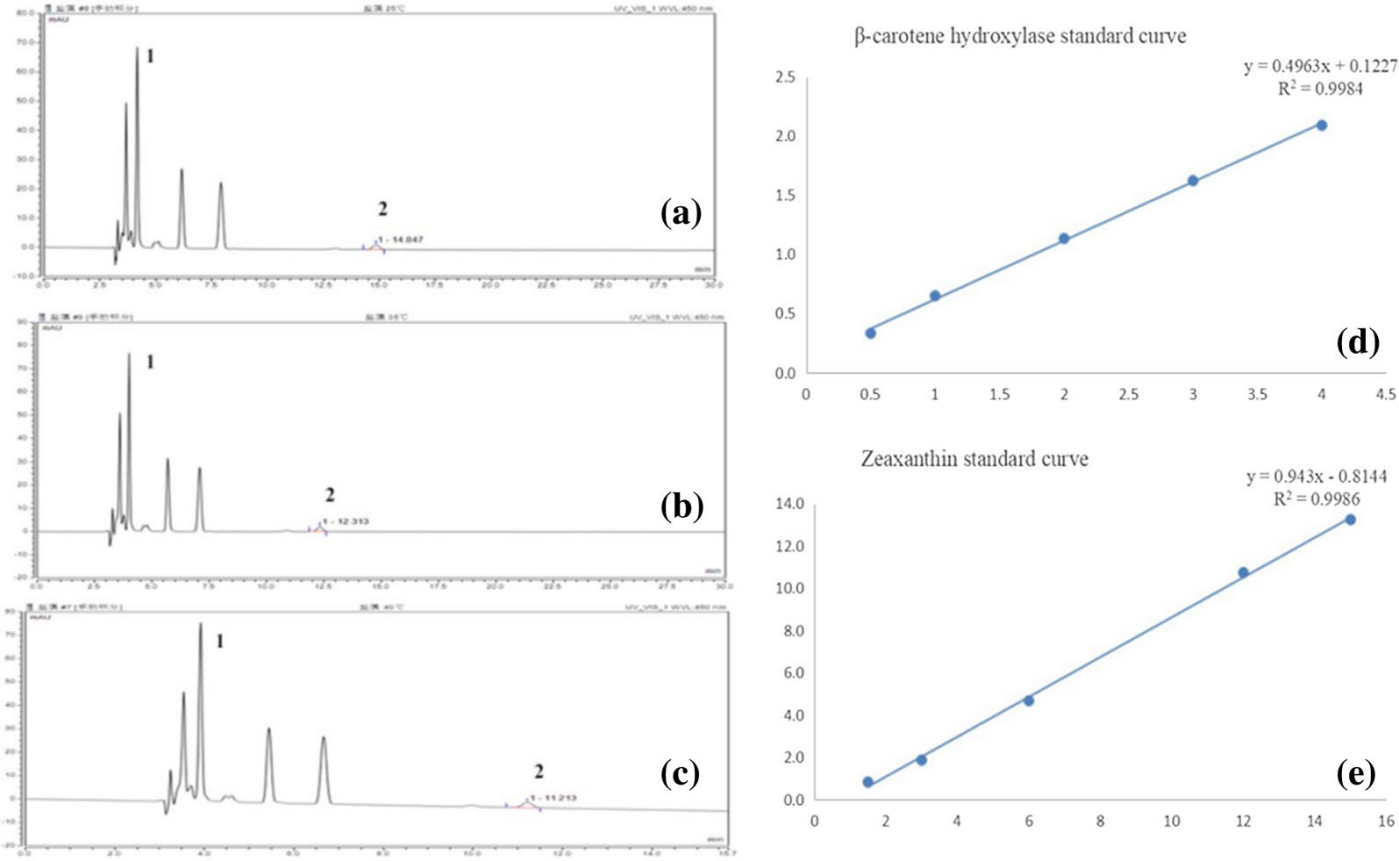
HPLC with diode-array detection and retention times of *D. salina* extracts at different temperatures. **a** HPLC chromatograms of WT *D. salina* extracts at 25 ℃ (Peak 1: zeaxanthin; Peak 2: β-carotene). **b** HPLC chromatograms of WT *D. salina* extracts at 35 ℃. **c** HPLC chromatograms of WT *D. salina* extracts at 40 ℃. **d** Standard curve of β-carotene. **e** Standard curve of zeaxanthin

**Fig.7:**
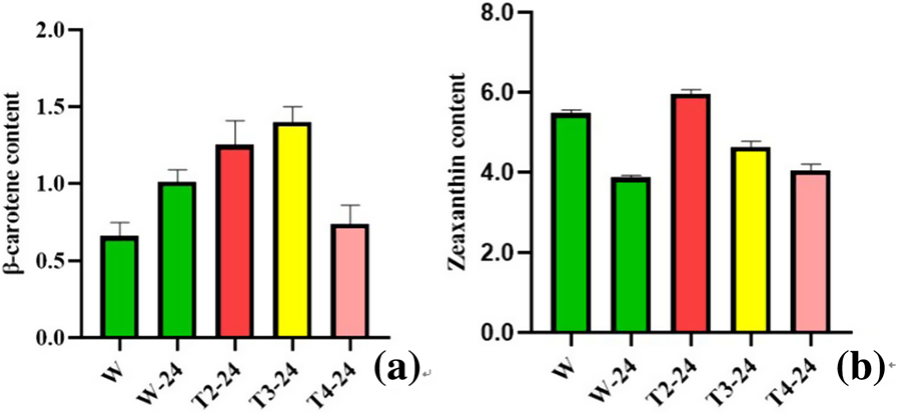
Content of β-carotene and zeaxanthin in *D. salina* after 24 h high light treatment (Values represented as mean ± SD (n = 3)). **a** The β-carotene content of WT strain and mutants after 24 h high light treatment. **b** The zeaxanthin content of WT strain and mutants after 24 h high light treatment

Before HPLC analysis, the various parameters were determined, including elution temperature, and mobile phase. Because *D. salina* sample has the largest peak area at 35 °C, 35 °C was used as the most suitable temperature for elution (Fig. 6b). After DAD monitoring at 450 nm, zeaxanthin and β-carotene profile showed two detectable peaks at 4, 12 min separately (Fig. 6a–c). Based on calibration curves of respective standards (Fig. 6d, e), the carotenoid content of *D. salina* was calculated refer to the experimental section. Through HPLC analysis, the total carotenoids content of three mutants were significantly different from those of WT strains (Fig. 7a). Under the 24 h light stress, both WT and mutant lines showed the accumulation of β-carotenoids. Compared with other lines, T3 line showed the maximum accumulation of β-carotenoids, up to 1.4 µg/mL. At the same time, there were significant changes in the level of downstream zeaxanthin (Fig. 7b). The amount of zeaxanthin in WT line was already significantly decreased after high light treatment for 24 h. Only T2 lines rose slightly, the other two mutant lines were down. All these results demonstrated that Dschyb genes were successfully knocked-out and the gene was phenotypically silenced.

## Discussion

Although *D. salina* is a promising host for production of foreign proteins, it still faces many challenges in practical application, including the unclear genome background, unavailability of effective molecular tools, the low or inconsistent expression of recombinant proteins, and so on (Liang et al. 2020). In our previous works, the expression of various exogenous genes in *D. salina* cells was transient, like the human canstatin, and viral envelope proteins (Zhang et al. 2018). To overcome these obstacles of *D. salina*, the emergence of CRISPR/Cas system offers an excellent integration point and a robust and precise genome editing tool for development of *D. salina* system*.* Through non-homologous end joining and homologues directed repair, CRISPR/Cas9 system provided a more direct route for targeted mutagenesis, causing a ablation or insertion of specific genomic sequences on a single step. Moreover, it has advantages of easy design, simplicity, specificity, and capability of multiplex genome editing. With the completion of chloroplast genome sequencing in *D. salina*, the development of chloroplast expression system will become an attractive research direction (https://www.ncbi.nlm.nih.gov/nuccore/GQ250046.1). Furthermore, using CRISPR technology to establish the nucleo-plasmic co-expression system of *D. salina* will become a more promising research direction, aiming to realize the high efficient production of exogenous genes.

Compared with the traditional transformation, RNP complex including preassembled Cas9 protein and sgRNA have enabled efficient genome editing in various hosts, such as animal, plants, human cells, and microalgae (DiNapoli et al. 2020; Xing et al. 2014; Kim et al. 2014; Liang et al. 2019). Owing to obviates the need for codon optimization or specific promoters, RNP delivery can be conveniently and rapidly applied in different species. Moreover, owing the degradation of Cas protein in cells by endogenous proteases, RNPs could reduce the off-target effects and mosaicism with a less cytotoxic in cells (Nomura et al. 2019). Simultaneously, the gene-edited plants and animals could be exempt from genetically modified regulations due to the absence of foreign DNA sequences (Kanchiswamy et al. 2015). Therefore, in order to further improve editing efficiency, our team will attempt to use RNP delivery system for gene editing of *D. salina* in next works. However, the disadvantages of RNP components need to be fully considered before the works, like the short half-life, and difficulty in transformation etc.

In conclusion, this study demonstrated that the specific-mutants of Dschyb have been successfully created with CRISPR/Cas9 system*.* After gene editing, Dschyb gene was knocked-out that induced a 2.2 fold increasing of β-carotene contents in mutant strains over than that of WT line. Owing to the first successful gene editing of *D. salina,* it has a very important practical significance for increasing carotene yield and meeting the realistic industry demand. Furthermore, this study not only provides an approach to overcome the current obstacles of *D. salina*, but also gives a strong tool to facilitates the development and application of *D. salina* system.

## Data Availability

The data of this article is included within the article. And also, the data and materials can also be requested from the corresponding author and the first author.

## Abbreviations

*D. salina*: *Dunaliella salina*
CRISPR: Clustered regularly interspaced short palindromic repeat
PCR: Polymerase chain reaction
HPLC: High-performance liquid chromatography
sgRNA: Single guide RNA
RT-qPCR: Real-time fluorescent quantitative PCR
Dschyb: Beta-carotene hydroxylase of *D. salina*
RNP: Ribonucleoprotein
LED: Light emitting diode
DAD: Diode array detector
WT: Wild type
